# Cluster Analysis of Global Research Hotspots and Thematic Evolution in Chronic Migraine: A Bibliometric Study (1917–2024)

**DOI:** 10.1155/prm/9934066

**Published:** 2026-08-20

**Authors:** Hongxu Chen, Qihui Chen, Yang Yang, Qijun Yu, Yonghui Pan, Jinghan Lin

**Affiliations:** ^1^ Department of Neurology, The First Affiliated Hospital of Harbin Medical University, Harbin 150001, China, hrbmu.edu.cn

**Keywords:** bibliometric analysis, chronic migraine, CiteSpace, global trends, VOSviewer

## Abstract

**Background:**

Chronic migraine is a prevalent and disabling neurological disorder with substantial impact on patients’ quality of life. Understanding the thematic structure and evolution of chronic migraine research is critical for guiding future studies and clinical practice. This study utilized cluster analysis to map research hotspots, thematic developments, and emerging trends in the global literature on chronic migraine.

**Methods:**

Relevant publications on chronic migraine from 1917 to 2024 were retrieved from the Web of Science Core Collection. Bibliometric and cluster analyses were performed using VOSviewer, CiteSpace, and R 4.3.3, focusing on keyword co‐occurrence, thematic clustering, and citation burst analysis to identify research hotspots and their evolution.

**Results:**

A total of 6366 articles were included. Cluster analysis of high‐frequency keywords identified five major research clusters: (1) Pathophysiological mechanisms and molecular targets, (2) epidemiology, comorbidities, and quality of life, (3) clinical trials and therapeutic strategies, (4) diagnosis and classification, and (5) regional and national burden. Thematic evolution analysis revealed a shift from early focus on epidemiology and symptomatology to recent emphasis on gene‐related peptides, preventive therapies, and resistance mechanisms. Burst keyword analysis highlighted emerging topics such as “calcitonin gene‐related peptide,” “preventive treatment,” and “central sensitization,” reflecting a growing interest in novel biologics and mechanisms of treatment resistance. The United States led in publication volume and collaboration, with *Headache*, *Cephalalgia*, and *Neurology* as core journals and Harvard University and the Mayo Clinic as the leading institutions.

**Conclusions:**

Cluster analysis identified five core research areas in chronic migraine and revealed a shift from epidemiological description to molecular mechanisms and individualized treatment. Future research is likely to focus on integrating pharmacological and non‐pharmacological interventions to further optimize patient outcomes.

## 1. Introduction

Chronic migraine is a widespread and severe neurological disorder characterized by headaches occurring on 15 or more days per month, with at least 8 days meeting the diagnostic criteria for migraine [1]. Chronic migraine typically involves a high frequency of attacks, with patients experiencing headaches for most of the month, in contrast to the less frequent episodes of intermittent migraine [2]. Epidemiological studies indicate that chronic migraine prevalence varies globally, but women are generally affected more than men, with the highest incidence observed in women aged between 20 and 40. Data show that the annual prevalence of chronic migraine is approximately 2%–4%, and its impact is substantially associated with marked disability and reduced quality of life [3]. Symptoms of chronic migraine include recurrent headaches that are usually unilateral but can also be bilateral. These headaches are often described as throbbing or pulsating and are frequently accompanied by nausea, vomiting, and heightened sensitivity to light and sound. The duration of these headaches typically ranges from 4 to 72 h, causing significant distress in patients’ lives. Attacks are often associated with triggers such as stress, sleep deprivation, specific foods and drinks (e.g., alcohol, caffeine, and chocolate), and hormonal fluctuations, particularly in women [4].

Although the exact pathophysiology of chronic migraine is not fully understood, research suggests that it is closely related to abnormalities in cerebrovascular function and excessive neural excitability. Specifically, chronic migraine may involve dysfunction in the brain’s pain modulation system and the brainstem. Imbalances in neurotransmitters, such as serotonin (5‐HT) and calcitonin gene‐related peptide (CGRP), are among the recognized mechanisms. Serotonin plays a crucial role in the neurotransmitter system, with its fluctuations linked to migraine occurrence. CGRP, a potent vasodilator, is associated with migraine attack frequency and severity [5]. The frequency and severity of chronic migraine attacks are influenced by several factors. Hormonal fluctuations are a significant factor in female patients, especially around the menstrual cycle, when migraines may occur more frequently. Stress, emotional changes, and sleep disorders are also known triggers, potentially provoking migraines by affecting the brain’s pain modulation mechanisms. Specific foods and beverages, such as alcohol, caffeine, and chocolate, are also considered potential triggers, as they can alter chemical balances in the body, inducing headaches [6, 7]. Management of chronic migraine involves a multifaceted approach, including pharmacological and non‐pharmacological strategies. Acute treatments typically involve medications such as non‐steroidal anti‐inflammatory drugs (NSAIDs) and triptans to alleviate acute attacks. Preventive medications, such as β‐adrenergic antagonists, calcium channel blockers, and anticonvulsants, are used to reduce the frequency and severity of attacks. Non‐pharmacological treatments include cognitive behavioral therapy, relaxation techniques, and biofeedback, which aim to reduce stress and improve lifestyle to decrease migraine frequency [8]. Recent research into chronic migraine treatment has advanced significantly, with new medications and therapies such as CGRP antagonists and neuromodulation techniques gradually entering clinical use. CGRP antagonists have shown promising results in treating chronic migraine, offering new hope for many patients. Neuromodulation techniques, such as transcranial magnetic stimulation (TMS) and vagus nerve stimulation (VNS), also demonstrate potential in reducing migraine attack frequency and severity. These emerging treatments provide additional options for managing chronic migraine and may alter the treatment landscape in the future.

In recent years, bibliometric analysis has become an essential approach for quantitatively mapping research trends, academic collaboration, and field development in chronic migraine studies [9]. However, previous analyses have rarely employed cluster analysis to systematically reveal the thematic structure, research hotspots, and their evolution within this field [10]. Cluster analysis, by identifying co‐occurring keywords and grouping them into major thematic clusters, provides deeper insights into the intellectual landscape and development trajectories of chronic migraine research. Therefore, this study utilizes cluster analysis as a central bibliometric method to comprehensively identify research hotspots, map thematic evolution, and uncover emerging trends in chronic migraine literature, with the goal of informing future research directions and promoting targeted resource allocation.

## 2. Materials and Methods

### 2.1. Literature Search and Screening

The data for this study were sourced from the Web of Science Core Collection (WoSCC). WoSCC was selected because it provides broad coverage of high‐impact international journals and includes standardized bibliographic records with complete cited‐reference information, which is essential for citation‐based analyses (e.g., co‐citation and collaboration network mapping) performed in this study. A literature search was conducted on May 29, 2024, covering publications from 1917 to 2024 related to chronic migraine. The search strategy was as follows: TS = (Chronic Migraine). To ensure robustness, we conducted pilot searches using related terms (e.g., “transformed migraine” and “chronic daily headache”); however, “chronic migraine” yielded the most relevant dataset for the scope of this analysis and minimized retrieval of non‐target headache entities. To avoid discrepancies due to ongoing database updates, all records were retrieved and exported on a single day in “Full Record and Cited References” format. Extracted fields included publication year, title, authors, affiliations/institutions, countries/regions, source journals, citations, references, and keywords. Keywords were derived from both Author Keywords and Keywords Plus (as provided by WoSCC) and were used for subsequent co‐occurrence, clustering, overlay, and burst analyses.

### 2.2. Statistical Analysis and Visualization

Three primary statistical analysis tools were used for data processing and visualization: VOSviewer (version 1.6.20), CiteSpace (version 6.3.R1), and R 4.3.3. Each tool served different analytical purposes. VOSviewer is a multifunctional bibliometric analysis tool used for institutional collaboration, author collaboration, co‐authorship, citation, and co‐citation analysis [11]. We utilized VOSviewer to analyze keyword co‐occurrence, exploring collaboration and relationship networks within the research field and revealing complex connections between authors, institutions, and publications [11]. To investigate emerging trends and research hotspots, CiteSpace was employed for keyword burst detection, using time slicing from January 1999 to May 2024 [12]. R 4.3.3 facilitated the visualization of publication outputs from corresponding authors across different countries and the article count from the top ten institutions.

We also used the H‐index to quantify the academic impact of individuals and journals [13]. The H‐index, a key metric for assessing researchers’ contributions and predicting future achievements, was sourced from WoSCC. Additionally, we analyzed Journal Citation Reports (JCR) (in 2023) quartiles and impact factors (IFs, in 2023) to further evaluate journal academic influence.

## 3. Results

**FIGURE 1:**
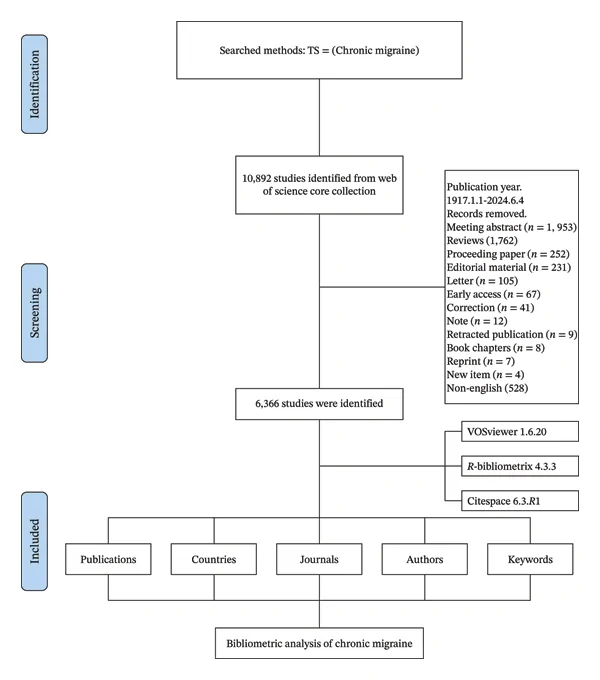
Flowchart of the literature screening process.

**FIGURE 2:**
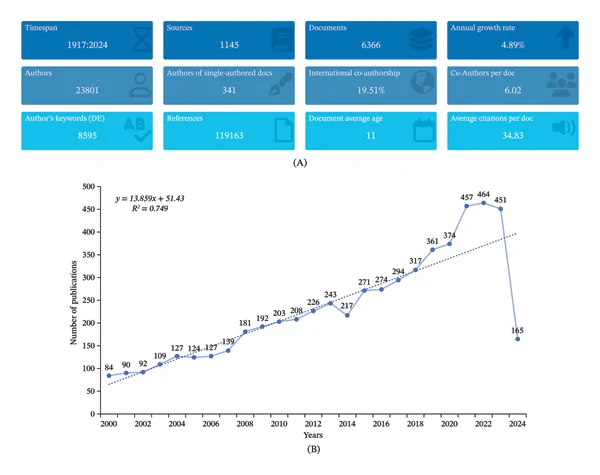
Overall overview and publication trends. (A) Overall information of publications. (B) Annual number of publications on chronic migraine.

In this study, the data selection process is illustrated in Figure 1. This study encompasses 6366 publications from 1145 sources spanning the years 1917 to 2024. These studies involved a total of 23,801 authors, with an average of 6.02 articles per author. Over this period, the average number of publications per year was 11, reflecting an annual growth rate of 4.89%. Collectively, these publications cited 119,163 references (Figure 2A).

### 3.1. An Overview of Publications in Research on Chronic Migraine

As shown in Figure 2B, the number of publications on the topic indicates a growing interest in this field from the years 2000 to 2023. Based on the annual growth rate of publications, the number of publications increased steadily after 2000—starting at around 80 per year and reaching nearly 500 by 2022, reflecting a growing interest in chronic migraine research (following the fitting function *y* = 13.859*x* + 51.43, *R* ^2^ = 0.749). This surge in publications may be attributed to advancements in technology, increased awareness, and heightened recognition of the impact of stress, all contributing to a greater focus on chronic migraine.

Additionally, we compiled a list of the most frequently cited articles in this field (Supporting Table 1). The most cited article is titled “Years lived with disability (YLDs) for 1160 sequelae of 289 diseases and injuries 1990–2010: A systematic analysis for the Global Burden of Disease Study 2010,” published in *The Lancet* (IF = 168.9) in 2012, and has been cited 4477 times [14].

### 3.2. Countries and Regions Analysis

**FIGURE 3:**
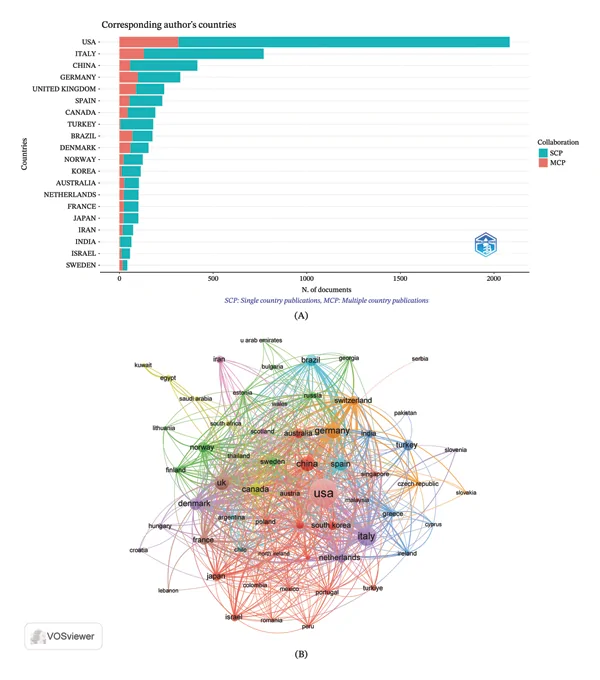
International distribution and collaboration of the corresponding author’s publications. (A) Distribution of corresponding author’s publications by country. (B) Visualization map depicting the collaboration among different countries.

According to Figure 3A and Supporting Table 2, the top 20 countries by publication count include the United States, Italy, China, and Germany, among others. The United States leads with 2084 publications (32.7%), followed by Italy (770 publications, 12.2%), China (416 publications, 6.6%), and Germany (325 publications, 5.1%). As a major research nation, the United States accounts for approximately 30% of the total publications, significantly surpassing other countries, especially Italy, which has over half the number of publications compared to the United States.

Single‐country publications (SCPs) dominate, suggesting that researchers often collaborate within their own countries. This trend may limit the field’s growth, as international collaborations (multiple country publications, MCP) are crucial for future research. European countries excel in MCP, with many having ratios above 0.3, reflecting the influence of political and geographical factors on medical research in Europe. Conversely, MCP ratios are relatively low among Asian countries, indicating a need for increased international collaboration in the region. From Figure 3B and Supporting Table 3, it is evident that the United States has research collaborations with almost every country, further consolidating its position as a global research powerhouse. European countries also have multiple collaboration networks, such as between Italy, the Netherlands, and Denmark, as well as between Germany and Switzerland. Additionally, Asian countries like China, South Korea, and Japan have established collaborations mainly with European countries (such as Belgium and Austria) and Australia, but the overall number of collaborations is still far less than that of the United States. These results suggest that, although geographical location is no longer a barrier to research collaboration, Asian countries still need to enhance their participation and influence in international projects to achieve more extensive and meaningful global cooperation. By strengthening multinational collaboration, significant progress is expected in the field of chronic migraine.

### 3.3. Journals and Co‐Cited Journals Analysis

A detailed analysis of journals related to chronic migraine research was conducted, focusing on impact metrics such as H‐index and IF. Detailed statistical data for these journals are provided in Supporting Table 4.

Among the top 10 journals by H‐index, the most influential are *Headache* (H‐index = 93), *Cephalalgia* (H‐index = 82), and *Neurology* (H‐index = 65). All are in JCR’s Q1 category, indicating their high academic impact and reputation. *Headache* leads with an H‐index of 93 and an IF of 5.0, reflecting its extensive citation and significance. *Cephalalgia* follows with an H‐index of 82 and an IF of 4.9, while *Neurology* has an H‐index of 65 and the highest IF among these, at 9.9. Among the top 20 journals by H‐index, *Brain* has the highest IF at 14.5, further validating its leading role in brain diseases and neuroscience.

**FIGURE 4:**
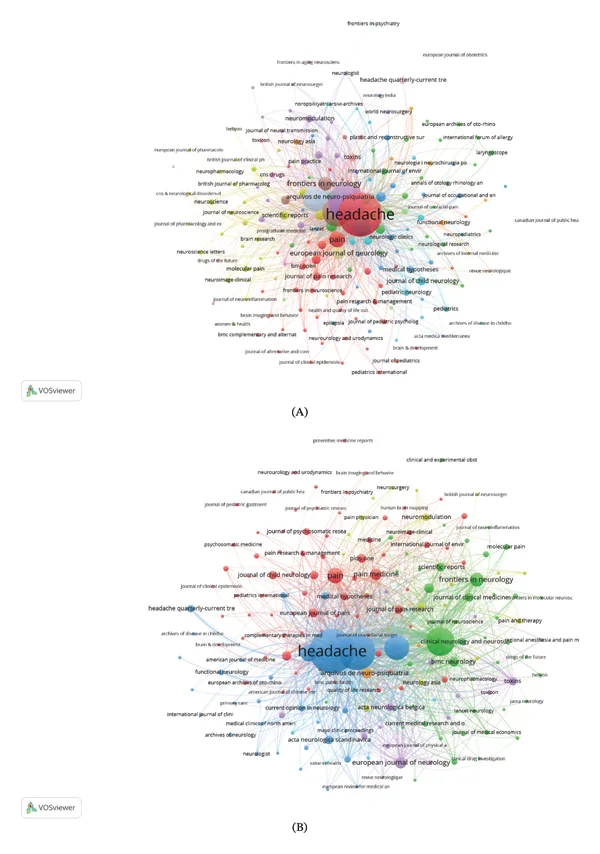
Journal co‐occurrence and coupling networks in chronic migraine research. (A) Co‐occurrence network of major journals publishing chronic migraine research, illustrating the relationships and thematic connections based on the frequency of co‐publication. (B) Coupling network analysis of these journals, highlighting the strength of citation links and collaborative patterns within the field.

To visualize the relationships between these journals, co‐occurrence (Figure 4A) and connection networks (Figure 4B) were created. In the co‐occurrence analysis, the three key journals with the highest total link strength were *Headache* (10,450), *Cephalalgia* (9804), and *Journal of Headache and Pain* (5164). In the connection networks, the top three journals with the highest total link strength were *Headache* (805,766), *Cephalalgia* (647,066), and *Journal of Headache and Pain* (454,347). The results show that *Headache*, *Cephalalgia*, and *Journal of Headache and Pain* are the top journals not only by link strength, confirming their central positions, but also by the high H‐index and IF in this field.

### 3.4. Author Impact Analysis

Among the top 10 authors by impact, all have an H‐index above 30 (Supporting Table 5). The author with the highest H‐index is Robert B. Lipton, with a total of 26,684 citations. Generally, there is a strong correlation between H‐index ranking and total citation count. However, this relationship is not absolute. For example, although the second‐highest citation count is attributed to Jan Olesen, with 20,507 citations, he does not appear in the top ten H‐index rankings. This discrepancy may be due to variations in the influence of the author’s affiliated institution and individual impact. Overall, five authors have a citation count exceeding 10,000, with two authors surpassing 20,000 citations.

**FIGURE 5:**
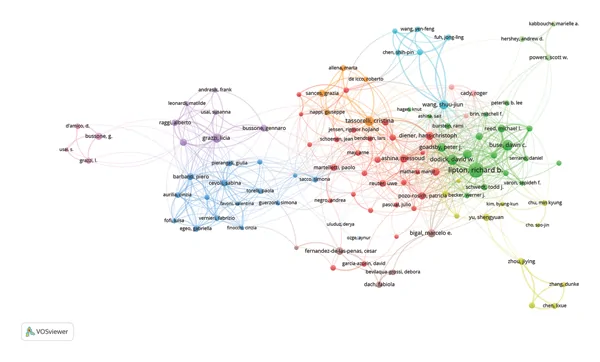
Visualization map depicting the collaboration among different authors.

To further explore author collaboration, a network of author‐related collaborations was constructed (Figure 5 and Supporting Tables 5, 6). Richard B. & Lipton has the highest number of collaborations with a total link strength of 408, followed by Down C. & Buse (250) and David W. & Dodick (190). The collaborations among authors are distinctly divided into several groups, which may be influenced by institutional affiliations and geographic locations. Total link strength was used to measure the degree of collaboration among authors, reflecting the intensity of cooperation between each node (i.e., author) and others in the network. A higher total link strength indicates closer collaboration. Notably, R.B. Lipton, the most‐cited author, is also among the most collaborative, with a total link strength of 408 and the highest number of publications (163).

From Figure 5, it is evident that author collaborations often occur within the same country, particularly among Chinese researchers. This collaboration pattern reflects the strong regional influence but may limit the diversity and innovation of international cooperation. While the close collaboration among Chinese researchers helps elevate domestic research standards, their lower involvement in international collaborations may constrain global perspectives and cross‐cultural exchange. Future efforts to enhance international collaboration, especially with other leading research countries, will be crucial for advancing global development in this field and addressing current research gaps.

### 3.5. Institutional Analysis

**FIGURE 6:**
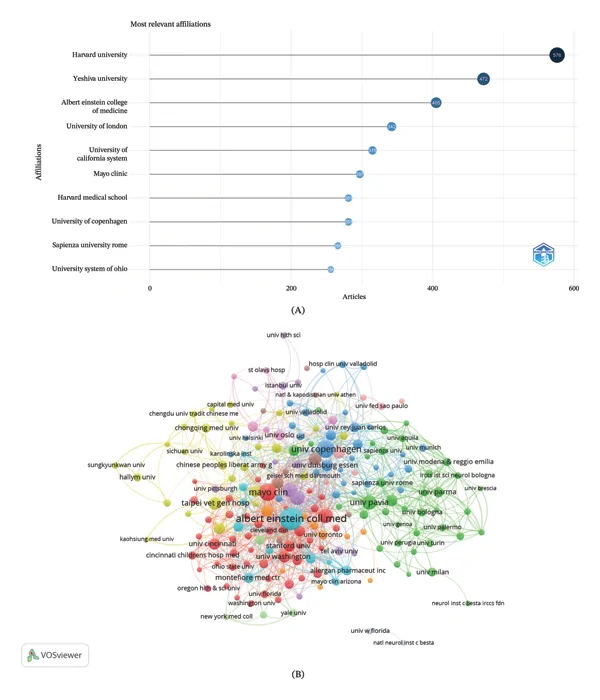
Analysis of institutional publication counts and collaborative networks. (A) Top ten institutions by article count and rank. (B) Visualization of collaboration among different institutions.

From the perspective of research institutions (Figure 6A), the top ten institutions are predominantly concentrated in two countries: The United States and Italy. The United States institutions occupy the top eight positions, with the only Italian institution, the University of Rome, ranking ninth. The University of Copenhagen in Denmark is positioned eighth. The three institutions with the highest number of related publications are Harvard University (576 articles, 9%), Yeshiva University (472 articles, 7%), and Albert Einstein College of Medicine (405 articles, 7%). These figures highlight the leadership and significant influence of the United States in the field of chronic migraine.

To better understand the collaboration among these institutions, a visualization of the 183 institutions with at least 15 publications was conducted, and a collaboration network was constructed (Figure 6B and Supporting Table 7). Among these institutions, Albert Einstein College of Medicine (684 collaborations) leads in international collaborations, followed by Mayo Clinic (352 collaborations) and the University of Copenhagen (279 collaborations). Despite Harvard University and Yeshiva University leading in publication volume, their international collaboration performance is not as prominent as that of Albert Einstein College of Medicine. This indicates that while Harvard and Yeshiva University are leading in numbers, their international collaboration projects are relatively fewer.

Although Mayo Clinic and the University of Copenhagen rank lower in publication volume, they excel in international collaboration, reflecting their significant roles in the global academic network. Both institutions rank among the top three in terms of collaborative projects, indicating their active participation in global academic exchange.

The collaboration network reveals that geographical location often influences cooperation, with considerable collaboration between the United States and European institutions. The top ten institutions in the United States have extensive networks with various research organizations, while European institutions, led by the University of Copenhagen, form closely connected networks. Asian institutions, such as Chongqing Medical University, Chengdu University of Traditional Chinese Medicine, and those in Taipei, play crucial roles but have relatively low international collaboration numbers. This geographically influenced collaboration model highlights the dominant roles of the United States and European institutions in chronic migraine research, while suggesting that Asian institutions need to enhance their international collaborations. This underscores the importance of global cooperation in advancing scientific research through resource and knowledge sharing.

### 3.6. Keyword and Citation Bursts

**FIGURE 7:**
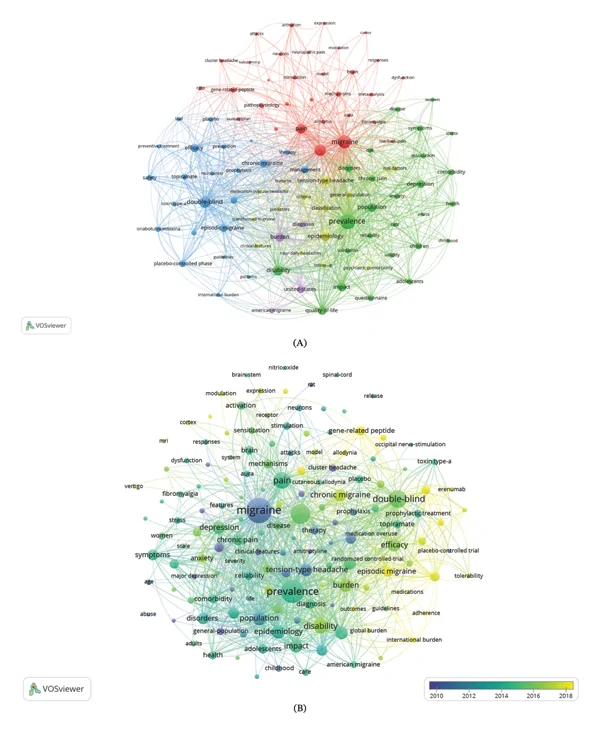
Visual analysis of keyword co‐occurrence network. (A) Keyword co‐occurrence network clusters. (B) Temporal overlay of keyword co‐occurrence network.

In analyzing the selected literature, both “Author Keywords” from the Bibliophagy application and “Keywords Plus” from the VOSviewer application were used. A total of 134 keywords were identified, each appearing at least 20 times. Supporting Table 8 helps identify research hotspots in the field. Keyword co‐occurrence mapping (Figure 7A) and subsequent cluster analysis revealed five distinct thematic clusters, each representing a central research focus: Cluster 1 (Red): Pathophysiological Mechanisms of Migraine. This cluster centers on the neurobiological underpinnings of migraine, highlighting terms such as “migraine,” “pain,” “gene‐related peptide,” “CGRP,” “central sensitization,” “allodynia,” “brain,” and “pathophysiology.” These keywords reflect the focus on molecular mechanisms, neural pathways, and the role of neuropeptides in migraine pathogenesis. Cluster 2 (Green): Epidemiology, Comorbidity, and Quality of Life. This group covers population‐level research, with emphasis on “prevalence,” “population,” “quality‐of‐life,” “disability,” “depression,” “anxiety,” “adolescents,” and “women.” The cluster underscores interest in the societal burden of migraine, comorbid conditions, and the impact on different demographic groups. Cluster 3 (Blue): Clinical Trials and Therapeutic Strategies. The keywords “double‐blind,” “efficacy,” “chronic migraine,” “preventive treatment,” “prophylaxis,” “episodic migraine,” and “placebo‐controlled phase” reflect robust research activity in clinical trial design, drug evaluation, and preventive therapy efficacy. Cluster 4 (Yellow): Diagnosis and Classification. This cluster is characterized by keywords such as “classification,” “diagnosis,” “clinical‐features,” “criteria,” “epidemiology,” and “tension‐type headache,” focusing on diagnostic criteria, differential diagnosis, and headache classification systems. Cluster 5 (Purple): Regional and National Burden. Comprising “american migraine,” “burden,” “diagnosis,” and “united‐states,” this small cluster highlights studies assessing the regional and national impact of migraine, especially within the United States.

**FIGURE 8:**
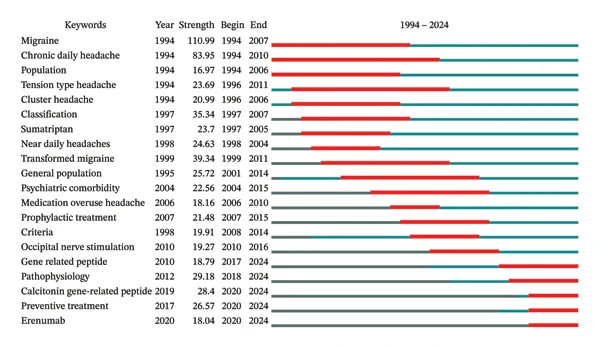
Top 20 keywords with the strongest citation bursts.

The overlay visualization (Figure 7B) arranges keywords along a timeline, reflecting their average year of appearance and the evolution of research priorities. Early‐stage studies (indicated by blue/purple nodes; ∼2010–2014) focused on foundational topics such as “migraine,” “pain,” “depression,” and “prevalence.” As the field progressed toward 2016–2018 (green to yellow nodes), newer keywords emerged, including “gene‐related peptide,” “erenumab,” and “prophylactic treatment,” highlighting a shift toward molecular targets, novel biologics, and advanced preventive therapies. This temporal pattern illustrates the dynamic progression from epidemiological and clinical characterization to cutting‐edge therapeutic innovation in chronic migraine research. Top 20 keywords with strong citation bursts identified by CiteSpace exhibited significant citation intensity and sustained interest (Figure 8). Each bar represents a year, with red bars indicating the intensity of the citation burst. The citation burst strength for these keywords ranges from 18 to 111, with most bursts lasting up to 5 years and some extending to 10 years, highlighting their ongoing impact and sustained attention. Since 2017, several keywords, including “gene related peptide,” “pathophysiology,” “calcitonin gene‐related peptide,” “preventive treatment,” and “erenumab,” have shown significant and continuing citation bursts. This concentration of bursts indicates a shift in research focus toward these areas and suggests potential future research trends and directions.

## 4. Discussion

### 4.1. Overall Research Results

This study provides a century‐spanning bibliometric and cluster‐based map of chronic migraine research (1917–2024), integrating keyword co‐occurrence clustering, overlay visualization, and burst detection to characterize how the field’s intellectual structure and priorities have evolved. Unlike narrative reviews that summarize clinical knowledge, our analysis focuses on how research activity is organized (major themes, influential contributors, and temporal shifts) and where new attention is concentrating. The sustained growth in annual publications—especially the pronounced acceleration after 2000—indicates increasing recognition of chronic migraine as a major cause of disability and a growing target for mechanistic and therapeutic innovation [15–17]. In parallel, the dominance of the United States in productivity and international collaboration, together with the visibility of major institutions, such as Harvard University and Yeshiva University, suggests that chronic migraine research is shaped by well‐established, resource‐intensive clinical and epidemiological infrastructures [9, 18]. Consistent with our journal network results, *Headache*, *Cephalalgia*, and *Neurology* remain central venues for disseminating influential chronic migraine studies, reflecting disciplinary consolidation and specialization [10].

A core contribution of this work is the identification of five stable, interpretable thematic clusters: (1) Pathophysiological mechanisms and molecular targets, (2) epidemiology/comorbidity/quality of life, (3) clinical trials and therapeutic strategies, (4) diagnosis/classification, and (5) regional/national burden—which together provide a structured “atlas” linking mechanistic discovery, clinical evaluation, and population impact. Importantly, the overlay and burst analyses indicate that the field has not progressed uniformly; rather, attention has shifted in stage‐like patterns from burden characterization toward targeted prevention and, more recently, toward mechanisms that may explain refractoriness and chronification.

### 4.2. Cluster 1 (Mechanisms and Molecular Targets): From Explanation to Actionable Targets

The concentration of high‐frequency and burst keywords around CGRP‐related terms and central sensitization indicates that mechanistic work has increasingly converged on pathways that are both biologically plausible and therapeutically tractable [19]. The prominence of “central sensitization” in more recent periods suggests that chronic migraine is increasingly conceptualized as a disorder of persistent neurobiological amplification rather than episodic symptom recurrence, which aligns with experimental emphasis on neuroimmune and glial mechanisms [20, 21]. In this context, the bibliometric prominence of CGRP‐targeted interventions is not merely reflective of clinical popularity; it represents a translational inflection point where a mechanistic hypothesis generated sustained, high‐impact clinical research streams [22]. At the same time, the appearance of resistant/refractory themes in later bursts supports the interpretation that the field is moving from “efficacy demonstration” to “response heterogeneity explanation,” motivating biomarker‐oriented stratification and mechanistic studies of non‐response [23].

### 4.3. Cluster 2 (Epidemiology, Comorbidity, and Quality of Life): Chronic Migraine as a Multidimensional Burden

This cluster’s persistence indicates that burden quantification and patient‐centered outcomes remain foundational drivers of the field. The frequent co‐occurrence of terms related to disability, depression, anxiety, sex differences, and population prevalence supports the view that chronic migraine research is strongly oriented toward real‐world impact, particularly in women and working‐age adults [15, 24]. From a bibliometric perspective, the prominence of these terms also helps explain why high‐citation papers often involve population studies and burden estimates: they provide shared reference points that connect mechanistic research to policy and resource allocation [24].

### 4.4. Cluster 3 (Clinical Trials and Therapeutic Strategies): Maturation of an Evidence‐Generating Ecosystem

The trial design and prevention‐related keyword structure reflects the growth of an intervention‐focused literature with increasingly standardized evaluation frameworks. The clustering of “double‐blind,” “placebo‐controlled,” and “preventive treatment” signals a mature RCT ecosystem in which pharmacologic prevention (including anti‐CGRP biologics) is evaluated alongside broader management strategies [22]. Notably, the co‐presence of behavioral and lifestyle‐related terms suggests increasing attention to multimodal care pathways rather than single‐intervention solutions [25, 26]. This pattern is consistent with clinical needs in chronic migraine, where comorbidities and long‐term management demands favor integrated strategies [25–27].

### 4.5. Cluster 4 (Diagnosis and Classification): Enabling Comparability and Reducing Misclassification

The diagnostic cluster underscores that research progress is constrained by definitional precision. The repeated appearance of classification and criteria‐related terms supports the continued centrality of standardized frameworks such as the International Classification of Headache Disorders (ICHD) for both clinical care and study comparability [28]. Bibliometrically, this cluster functions as a “methodological backbone”: without reliable case definitions, mechanistic studies risk heterogeneity, and trials risk outcome dilution. The continuing linkage with tension‐type headache and related terms highlights that misclassification remains a practical and research‐relevant barrier [29].

### 4.6. Cluster 5 (Regional and National Burden): Translating Evidence Into Health‐System Priorities

Although smaller in the keyword network, this cluster is important because it anchors chronic migraine research within health‐system and policy contexts. Highly cited burden work (e.g., Global Burden of Disease [GBD]‐related outputs) provides quantifiable arguments for prevention investment, access to effective therapies, and patient education [24]. The cluster’s presence also highlights an equity‐relevant gap: Burden is global, but research visibility and infrastructure remain concentrated, reinforcing the importance of broader international participation [30].

### 4.7. Thematic Evolution: What the Overlay and Burst Results Add Beyond Cluster Labels

The overlay and burst analyses refine the interpretation of “hotspots” by showing when topics gained momentum and how priorities shifted. Early periods emphasize foundational constructs (pain, prevalence, depression), which aligns with the need to define and quantify chronic migraine as a public health problem [3–5, 17]. Later, bursts concentrated around CGRP, and gene‐related peptide terms mark the transition to target‐driven prevention research and the clinical translation of novel biologics [19, 22, 31]. Most recently, the persistence of bursts for preventive treatment and central sensitization, alongside the increased attention to resistance/refractory themes, suggests a field moving toward solving second‐order problems: why some patients fail to respond, how chronification is maintained, and how long‐term management can be optimized [23]. This “evolutionary signature” is a key value of the bibliometric approach: it demonstrates that the current frontier is not simply discovering new therapies but improving durability, personalization, and mechanistic understanding of non‐response.

### 4.8. Non‐Pharmacological Interventions: Meditation and Mindfulness (2020–2023)

Recent years have seen a surge of interest in non‐drug interventions, particularly meditation and mindfulness‐based stress reduction (MBSR), as highlighted by keyword bursts such as “meditation,” “MBSR,” and “quality‐of‐life.” Randomized controlled trials and meta‐analyses have demonstrated that these interventions can reduce migraine frequency, lessen pain intensity, and improve emotional well‐being [32, 33]. These therapies modulate the autonomic nervous system, reduce stress, and bolster emotional regulation, offering valuable adjuncts or alternatives for patients with suboptimal responses to pharmacotherapy [34]. The integration of these approaches into routine care is consistent with current recommendations for holistic, patient‐centered management of chronic migraine.

### 4.9. Treatment Resistance and Central Sensitization: Future Frontiers (2023 and Beyond)

Emerging research themes, as revealed by recent citation bursts, include “resistance mechanisms” and “central sensitization.” Despite advances in CGRP‐targeted therapies, a subset of patients experiences inadequate or diminishing responses, underlining the urgent need to elucidate resistance pathways and identify predictive biomarkers [23, 35]. Psychological comorbidities such as depression and anxiety further complicate treatment, supporting the necessity for integrated, multidisciplinary management [27]. Experimental studies have identified neuroimmune mechanisms—such as TREM1‐mediated NF‐κB activation, adenosine monophosphate–activated protein kinase (AMPK) signaling, hippocampal HCN2 channels, and N‐methyl‐D‐aspartate receptor (NMDA) receptor subunits—as key contributors to central sensitization and migraine chronification [20, 21, 36]. Advanced neuroimaging and electrophysiological studies are now beginning to clarify the thalamocortical and cortical inhibitory deficits underlying medication overuse headache (MOH) and refractory migraine, paving the way for both diagnostic and therapeutic innovation [37].

Taken together, our bibliometric findings suggest three practical implications. First, the strong linkage between mechanistic terms (CGRP, central sensitization) and trial terms (preventive treatment, double‐blind RCTs) indicates that chronic migraine is one of the headache domains where translational pathways are functioning—mechanisms are generating testable interventions at scale [19, 22]. Second, the sustained prominence of comorbidity and quality‐of‐life keywords supports the need for care models that integrate mental health screening and behavioral interventions into prevention strategies, rather than treating them as optional adjuncts [27]. Third, the continued visibility of diagnosis/classification terms highlights that future innovation will depend on improving phenotyping and reducing misclassification across clinical settings, which is essential for both real‐world effectiveness and research reproducibility [38, 39].

### 4.10. Future Outlook

Looking forward, chronic migraine research is expected to increasingly focus on unraveling the molecular and network‐level basis of migraine refractoriness and chronification. Personalized medicine approaches—leveraging biomarkers, neuroimaging, and digital phenotyping—will likely become central to research and clinical care. The integration of pharmacological, behavioral, and neuromodulatory therapies, guided by mechanistic insights, is poised to define the next era of chronic migraine management. Addressing medication overuse and understanding mechanisms of treatment resistance remain critical priorities [37, 40, 41], with future studies needed to develop early identification tools, optimize patient education, and refine combination or sequential therapy strategies.

### 4.11. Strengths and Limitations

This study’s strengths include the use of a large, high‐quality dataset and advanced bibliometric tools, enabling a nuanced analysis of research trends, influential entities, and thematic evolution over more than a century. Our approach offers actionable insights for researchers, clinicians, and policymakers.

However, several limitations should be acknowledged. First, reliance on the WoSCC may have resulted in the omission of relevant literature from non‐English or less prominent journals, potentially introducing selection bias. Second, bibliometric analyses are inherently retrospective and may not fully capture the most recent developments or emerging research directions. Third, the interpretation of keyword clustering and citation bursts is subject to the limitations of automated text mining and may not reflect the full complexity of the scientific discourse. Finally, while we highlight global trends, regional disparities and the unique needs of low‐ and middle‐income countries warrant further qualitative investigation.

Future bibliometric studies should incorporate additional databases, employ mixed‐methods approaches, and provide ongoing updates to capture the rapidly evolving landscape of migraine research.

This bibliometric analysis systematically charts the global landscape and thematic evolution of chronic migraine research over the past century. The field has shifted from descriptive and symptom‐based approaches to a deeper focus on molecular mechanisms, particularly CGRP, central sensitization, and psychological comorbidities. Despite notable advances, challenges remain in addressing treatment resistance, diagnostic complexity, and unequal access to care. Future progress will depend on precision medicine, improved biomarkers, integrative therapies, and strengthened international collaboration to ensure more effective and equitable management of chronic migraine.

## Author Contributions

(I) Conception and design: Yonghui Pan.

(II) Administrative support: Hongxu Chen and Qihui Chen.

(III) Data analysis and interpretation: Qijun Yu and Yang Yang.

(IV) Manuscript writing and final approval of manuscript: Jinghan Lin.

## Funding

The study was supported by the First Affiliated Hospital of Harbin Medical University, which funded the training of young medical talents 2024YQ22, Heilongjiang Province Postdoctoral Research Start‐Up Fund 21042240057, Heilongjiang Province Natural Science Foundation Key Research and Development project 2023ZX06C02, and National Natural Science Foundation project 82071549.

## Ethics Statement

The authors have nothing to report.

## Consent

The authors have nothing to report.

## Conflicts of Interest

The authors declare no conflicts of interest.

## Supporting Information

Additional supporting information can be found online in the Supporting Information section.

## Supporting information


**Supporting Information** Supporting Table 1 The top 50 most cited articles. Supporting Table 2 Publication and citation profiles of leading countries. Supporting Table 3 Total link strength of leading countries. Supporting Table 4 Bibliometric indicators of high‐impact journals. Supporting Table 5 Publication and citation profiles of high‐impact authors. Supporting Table 6 Author collaboration statistics. Supporting Table 7 Institution publication statistics. Supporting Table 8 Keyword co‐occurrence network analysis.

## Data Availability

All data generated or analyzed during this study are included in this published article.
