# Supplementary material for: Cluster Analysis of Global Research Hotspots and Thematic Evolution in Chronic Migraine: A Bibliometric Study (1917–2024)
Source: Pain Res Manag. 2026 Aug 20;2026:9934066. doi: 10.1155/prm/9934066 (PMC13494518; doi:10.1155/prm/9934066)
Supplement: Supplementary file 1 [file PRM-2026-9934066-s001.docx]

**Supplementary Table 1** The Top 50 Most Cited Articles

| Paper | DOI | Total Citations | TC per Year | Normalized TC |
| --- | --- | --- | --- | --- |
| VOS T, 2012, LANCET | 10.1016/S0140-6736(12)61729-2 | 4477 | 344.38 | 78.54 |
| OLESEN J, 2018, CEPHALALGIA | 10.1177/0333102417738202 | 4338 | 619.71 | 115.41 |
| BES A, 2013, CEPHALALGIA | 10.1177/0333102413485658 | 3208 | 267.33 | 63.23 |
| TORELLI P, 2004, CEPHALALGIA | 10.1111/j.1468-2982.2004.00633.x | 2932 | 139.62 | 35.17 |
| VOS T, 2016, LANCET | 10.1016/s0140-6736(16)31678-6 | 2712 | 301.33 | 66.57 |
| AARONSON NK, 1998, J CLIN EPIDEMIOL | 10.1016/S0895-4356(98)00097-3 | 1763 | 65.3 | 20.96 |
| VOS T, 2017, LANCET | 10.1016/S0140-6736(17)32154-2 | 1440 | 180 | 38.61 |
| MAY A, 1998, LANCET | 10.1016/S0140-6736(98)02470-2 | 802 | 29.7 | 9.54 |
| LUND JP, 1991, CAN J PHYSIOL PHARM | 10.1139/y91-102 | 762 | 22.41 | 10.84 |
| COKER AL, 2000, ARCH FAM MED | 10.1001/archfami.9.5.451 | 731 | 29.24 | 9.99 |
| OLESEN J, 2006, CEPHALALGIA | 10.1111/j.1468-2982.2006.01172.x | 729 | 38.37 | 11.7 |
| HARMON KG, 2013, BRIT J SPORT MED | 10.1136/bjsports-2012-091941 | 713 | 59.42 | 14.05 |
| DIENER HC, 2010, CEPHALALGIA | 10.1177/0333102410364677 | 677 | 45.13 | 13.23 |
| HIPPISLEY-COX J, 2017, BMJ-BRIT MED J | 10.1136/bmj.j2099 | 675 | 84.38 | 18.1 |
| AURORA SK, 2010, CEPHALALGIA | 10.1177/0333102410364676 | 619 | 41.27 | 12.1 |
| DODICK DW, 2010, HEADACHE | 10.1111/j.1526-4610.2010.01678.x | 612 | 40.8 | 11.96 |
| PERQUIN CW, 2000, PAIN | 10.1016/S0304-3959(00)00269-4 | 610 | 24.4 | 8.33 |
| ROTH-ISIGKEIT A, 2005, PEDIATRICS | 10.1542/peds.2004-0682 | 578 | 28.9 | 8.97 |
| BIGAL ME, 2008, HEADACHE-a | 10.1111/j.1526-4610.2008.01217.x | 563 | 33.12 | 11.15 |
| KELMAN L, 2007, CEPHALALGIA | 10.1111/j.1468-2982.2007.01303.x | 539 | 29.94 | 8.62 |
| SCHER AI, 2003, PAIN | 10.1016/S0304-3959(03)00293-8 | 505 | 22.95 | 9.21 |
| TEPPER S, 2017, LANCET NEUROL | 10.1016/S1474-4422(17)30083-2 | 491 | 61.38 | 13.17 |
| SILBERSTEIN SD, 2017, NEW ENGL J MED | 10.1056/NEJMoa1709038 | 480 | 60 | 12.87 |
| MCQUAY H, 1995, BRIT MED J | 10.1136/bmj.311.7012.1047 | 474 | 15.8 | 9.38 |
| SCHWARTZ BS, 1998, JAMA-J AM MED ASSOC | 10.1001/jama.279.5.381 | 456 | 16.89 | 5.42 |
| PIECZENIK SR, 2007, EXP MOL PATHOL | 10.1016/j.yexmp.2006.09.008 | 452 | 25.11 | 7.23 |
| LINDE K, 2005, JAMA-J AM MED ASSOC | 10.1001/jama.293.17.2118 | 447 | 22.35 | 6.93 |
| BLUMENFELD AM, 2011, CEPHALALGIA | 10.1177/0333102410381145 | 445 | 31.79 | 9.9 |
| CASTILLO J, 1999, HEADACHE | 10.1046/j.1526-4610.1999.3903190.x | 440 | 16.92 | 9.62 |
| SCHER AI, 1998, HEADACHE | 10.1046/j.1526-4610.1998.3807497.x | 438 | 16.22 | 5.21 |
| AYATA C, 2006, ANN NEUROL | 10.1002/ana.20778 | 430 | 22.63 | 6.9 |
| WELCH KMA, 2001, HEADACHE | 10.1046/j.1526-4610.2001.041007629.x | 430 | 17.92 | 7.25 |
| BUSE DC, 2010, J NEUROL NEUROSUR PS | 10.1136/jnnp.2009.192492 | 422 | 28.13 | 8.25 |
| ONGHENA P, 1992, PAIN | 10.1016/0304-3959(92)90144-Z | 422 | 12.79 | 8.05 |
| HEMP P, 2004, HARVARD BUS REV | NA | 416 | 19.81 | 4.99 |
| LINDE K, 2007, PAIN | 10.1016/j.pain.2006.12.006 | 408 | 22.67 | 6.52 |
| DIENER HC, 2007, CEPHALALGIA | 10.1111/j.1468-2982.2007.01326.x | 407 | 22.61 | 6.51 |
| HUDSON JI, 1992, AM J MED | 10.1016/0002-9343(92)90265-D | 406 | 12.3 | 7.75 |
| COLLINS JJ, 2005, J OCCUP ENVIRON MED | 10.1097/01.jom.0000166864.58664.29 | 385 | 19.25 | 5.97 |
| STEWART WF, 2000, PAIN | 10.1016/S0304-3959(00)00305-5 | 380 | 15.2 | 5.19 |
| RODRIGUEZ-RAECKE R, 2009, J NEUROSCI | 10.1523/JNEUROSCI.3687-09.2009 | 378 | 23.63 | 8.73 |
| BIGAL ME, 2008, NEUROLOGY | 10.1212/01.wnl.0000323925.29520.e7 | 375 | 22.06 | 7.42 |
| MATHARU MS, 2004, BRAIN | 10.1093/brain/awh022 | 371 | 17.67 | 4.45 |
| SILBERSTEIN SD, 2007, HEADACHE | 10.1111/j.1526-4610.2006.00684.x | 364 | 20.22 | 5.82 |
| SILBERSTEIN SD, 1994, HEADACHE | 10.1111/j.1526-4610.1994.hed3401001.x | 357 | 11.52 | 6.75 |
| BUSE DC, 2012, HEADACHE | 10.1111/j.1526-4610.2012.02223.x | 352 | 27.08 | 6.17 |
| SIMPSON DM, 2016, NEUROLOGY | 10.1212/WNL.0000000000002560 | 351 | 39 | 8.62 |
| DETKE HC, 2018, NEUROLOGY | 10.1212/WNL.0000000000006640 | 341 | 48.71 | 9.07 |
| VONKORFF M, 1996, BRIT J PSYCHIAT | 10.1192/S0007125000298474 | 336 | 11.59 | 6.24 |
| ALLEN JH, 2004, GUT | 10.1136/gut.2003.036350 | 335 | 15.95 | 4.02 |

**Supplementary Table 2** Publication and Citation Profiles of Leading Countries

| **Country** | **Articles** | **Freq** | **MCP_Ratio** | **TP** | **TP_rank** | **TC** | **TC_rank** | **Average Citations** |
| --- | --- | --- | --- | --- | --- | --- | --- | --- |
| United States | 2,084 | 0.327 | 0.151 | 7,758 | 1 | 93,928 | 1 | 45.1 |
| Italy | 770 | 0.121 | 0.170 | 2,888 | 2 | 20,951 | 2 | 27.2 |
| China | 416 | 0.065 | 0.135 | 1,773 | 3 | 8,110 | 5 | 19.5 |
| Germany | 325 | 0.051 | 0.302 | 1,145 | 4 | 12,733 | 4 | 39.2 |
| United Kingdom | 239 | 0.038 | 0.368 | 1,079 | 6 | 15,666 | 3 | 65.5 |
| Spain | 229 | 0.036 | 0.227 | 1,137 | 5 | 5,581 | 9 | 24.4 |
| Canada | 192 | 0.030 | 0.224 | 771 | 7 | 7,152 | 6 | 37.2 |
| Turkey | 180 | 0.028 | 0.028 | 564 | 9 | 2,684 | 13 | 14.9 |
| Brazil | 177 | 0.028 | 0.390 | 594 | 8 | 3,981 | 11 | 22.5 |
| Denmark | 156 | 0.025 | 0.378 | 498 | 12 | 7,136 | 7 | 45.7 |
| Norway | 125 | 0.020 | 0.168 | 557 | 10 | 4,535 | 10 | 36.3 |
| South Korea | 114 | 0.018 | 0.096 | 468 | 15 | 1,906 | 15 | 16.7 |
| Australia | 104 | 0.016 | 0.250 | 492 | 13 | 3,173 | 12 | 30.5 |
| Netherlands | 102 | 0.016 | 0.216 | 469 | 14 | 6,363 | 8 | 62.4 |
| France | 101 | 0.016 | 0.228 | 501 | 11 | 2,512 | 14 | 24.9 |
| Japan | 101 | 0.016 | 0.198 | 381 | 16 | 1,227 | 19 | 12.1 |
| Iran | 73 | 0.011 | 0.247 | 358 | 17 | 1,116 | 20 | 15.3 |
| India | 64 | 0.010 | 0.047 | 216 | 20 | 912 | 21 | 14.2 |
| Israel | 57 | 0.009 | 0.158 | 217 | 19 | 1,288 | 18 | 22.6 |
| Sweden | 42 | 0.007 | 0.429 | 194 | 21 | 1,553 | 17 | 37 |

Note(s): Articles: Publications of Corresponding Authors only. Freq: Frequence of Total Publications. MCP_Ratio: Proportion of Multiple Country Publications. TP: Total Publications. TP_rank: Rank of Total Publications. TC: Total Citations. TC_rank: Rank of Total Citations. Average Citations: The average number of citations per publication.

**Supplementary Table 3** Total Link Strength of Leading Countries

| **Rank** | **ID** | **Country** | **Documents** | **Citations** | **Total Link Strength** |
| --- | --- | --- | --- | --- | --- |
| 1 | 95 | United States | 2450 | 98,837 | 28,521 |
| 2 | 40 | Italy | 885 | 24,356 | 14,528 |
| 3 | 27 | Germany | 478 | 21,323 | 11,767 |
| 4 | 92 | United Kingdom | 425 | 23,295 | 9,169 |
| 5 | 19 | Denmark | 262 | 14,664 | 8,525 |
| 6 | 81 | Spain | 318 | 8,488 | 7,015 |
| 7 | 63 | People's Republic of China | 453 | 9,170 | 5,035 |
| 8 | 11 | Canada | 288 | 11,732 | 3,532 |
| 9 | 56 | Netherlands | 177 | 9,832 | 3,336 |
| 10 | 60 | Norway | 163 | 5,591 | 2,972 |
| 11 | 8 | Brazil | 218 | 4,940 | 2,862 |
| 12 | 25 | France | 135 | 4,041 | 2,103 |
| 13 | 86 | Switzerland | 115 | 3,883 | 2,096 |
| 14 | 80 | South Korea | 130 | 2,292 | 1,970 |
| 15 | 89 | Turkey | 193 | 3,324 | 1,910 |
| 16 | 6 | Belgium | 87 | 3,491 | 1,909 |
| 17 | 3 | Australia | 144 | 4,359 | 1,350 |
| 18 | 85 | Sweden | 88 | 3,248 | 1,204 |
| 19 | 42 | Japan | 123 | 1,724 | 1,169 |
| 20 | 29 | Greece | 45 | 909 | 1,160 |

**Supplementary Table 4** Bibliometric Indicators of High-Impact Journals

| **Journal** | **H_**  **index** | **IF** | **JCR_**  **Quartile** | **PY_**  **start** | **TP** | **TP_**  **rank** | **TC** | **TC_**  **rank** |
| --- | --- | --- | --- | --- | --- | --- | --- | --- |
| *Headache* | 93 | 5.0 | 1 | 1980 | 934 | 1 | 25,789 | 2 |
| *Cephalalgia* | 82 | 4.9 | 1 | 1985 | 626 | 2 | 26,634 | 1 |
| *Neurology* | 65 | 9.9 | 1 | 1951 | 141 | 5 | 10,911 | 3 |
| *Pain* | 55 | 7.4 | 1 | 1980 | 121 | 6 | 7,930 | 4 |
| *Journal of Headache and Pain* | 54 | 7.4 | 1 | 2007 | 391 | 3 | 7,903 | 5 |
| *Neurological Sciences* | 31 | 3.3 | 2 | 2003 | 290 | 4 | 2,065 | 10 |
| *European Journal of Neurology* | 27 | 5.1 | 1 | 1997 | 66 | 8 | 1,843 | 12 |
| *Journal of Neurology* | 23 | 6.0 | 1 | 1977 | 48 | 11 | 987 | 21 |
| *Clinical Journal of Pain* | 22 | 2.9 | 2 | 1991 | 40 | 13 | 1,045 | 20 |
| *Pain Medicine* | 22 | 3.1 | 2 | 2002 | 62 | 9 | 760 | 29 |
| *European Journal of Pain* | 21 | 3.6 | 2 | 2002 | 38 | 14 | 811 | 26 |
| *Brain* | 20 | 14.5 | 1 | 1995 | 27 | 25 | 2,142 | 8 |
| *Frontiers in Neurology* | 19 | 3.4 | 2 | 2012 | 92 | 7 | 655 | 38 |
| *Journal of Pain* | 18 | 4.0 | 2 | 2002 | 33 | 22 | 1,256 | 18 |
| *PLOS ONE* | 17 | 3.7 | 2 | 2010 | 38 | 16 | 1,294 | 17 |
| *Acta Neurologica Scandinavica* | 16 | 3.5 | 2 | 1962 | 34 | 19 | 297 | 99 |
| *Current Opinion in Neurology* | 16 | 4.8 | 1 | 1995 | 21 | 33 | 794 | 28 |
| *Journal of Neurology, Neurosurgery & Psychiatry* | 16 | 11.0 | 1 | 1984 | 19 | 39 | 1,740 | 14 |
| *Pediatrics* | 16 | 8.0 | 1 | 1995 | 16 | 51 | 510 | 51 |
| *Journal of Child Neurology* | 15 | 1.9 | 4 | 1994 | 34 | 20 | 286 | 105 |

Note(s): H_index: The h-index of the journal, which measures both the productivity and citation impact of the publications. IF: Impact Factor, indicating the average number of citations to recent articles published in the journal. JCR_Quartile: The quartile ranking of the journal in the Journal Citation Reports, indicating the journal's ranking relative to others in the same field (Q1: top 25%, Q2: 25%-50%, Q3: 50%-75%, Q4: bottom 25%). TP: Total Publications. TP_rank: Rank of Total Publications. TC: Total Citations. TC_rank: Rank of Total Citations. Average Citations: The average number of citations per publication. PY_start: Publication Year Start, indicating the year the journal started publication.

**Supplementary Table 5** Publication and Citation Profiles of High-Impact Authors

| **Authors** | **H_index** | **g-index** | **m-index** | **PY_start** | **TP** | **TP_Frac** | **TP_rank** | **TC** | **TC_rank** |
| --- | --- | --- | --- | --- | --- | --- | --- | --- | --- |
| Lipton RB | 78 | 161 | 2.167 | 1989 | 221 | 0.46 | 1 | 26,684 | 1 |
| Goadsby PJ | 51 | 94 | 1.500 | 1991 | 94 | 0.24 | 5 | 15,846 | 3 |
| Silberstein SD | 48 | 106 | 1.412 | 1991 | 106 | 0.29 | 2 | 12,752 | 4 |
| Bigal ME | 44 | 83 | 1.913 | 2002 | 90 | 0.22 | 7 | 7,004 | 11 |
| Diener HC | 42 | 80 | 1.167 | 1989 | 80 | 0.13 | 10 | 10,834 | 5 |
| Dodick DW | 40 | 86 | 1.600 | 2000 | 96 | 0.18 | 4 | 7,475 | 10 |
| Buse DC | 38 | 75 | 2.375 | 2009 | 89 | 0.12 | 8 | 5,704 | 12 |
| Aurora SK | 32 | 49 | 1.333 | 2001 | 49 | 0.09 | 19 | 5,462 | 15 |
| Jensen R | 32 | 43 | 0.941 | 1991 | 43 | 0.11 | 29 | 9,291 | 7 |
| Olesen J | 32 | 50 | 0.711 | 1980 | 50 | 0.17 | 18 | 20,507 | 2 |
| Wang SJ | 31 | 86 | 1.148 | 1998 | 86 | 0.14 | 9 | 9,620 | 6 |
| Tepper SJ | 30 | 48 | 1.250 | 2001 | 66 | 0.17 | 13 | 2,444 | 19 |
| Reed ML | 28 | 45 | 1.400 | 2005 | 45 | 0.06 | 25 | 3,150 | 18 |
| Fuh JL | 27 | 42 | 1.038 | 1999 | 42 | 0.08 | 30 | 5,555 | 13 |
| Jensen RH | 27 | 45 | 2.077 | 2012 | 50 | 0.10 | 17 | 2,028 | 22 |
| Nappi G | 27 | 54 | 0.730 | 1988 | 54 | 0.07 | 14 | 9,110 | 8 |
| Bendtsen L | 26 | 41 | 0.897 | 1996 | 41 | 0.09 | 31 | 5,169 | 16 |
| Bussone G | 26 | 46 | 0.650 | 1985 | 92 | 0.20 | 6 | 2,436 | 20 |
| Grazzi L | 26 | 38 | 1.130 | 2002 | 103 | 0.24 | 3 | 1,942 | 23 |
| Katsarava Z | 25 | 47 | 1.190 | 2004 | 47 | 0.07 | 22 | 5,487 | 14 |

Note(s): H_index: The h-index of the journal, which measures both the productivity and citation impact of the publications. g_index: The g-index of the journal, which gives more weight to highly-cited articles. m_index: The m-index of the journal, which is the h-index divided by the number of years since the first published paper. TP: Total Publications. TP_rank: Rank of Total Publications. TC: Total Citations. TC_rank: Rank of Total Citations. Average Citations: The average number of citations per publication. PY_start: Publication Year Start, indicating the year the journal started publication.

**Supplementary Table 6** Author Collaboration Statistics

| **ID** | **Author** | **Documents** | **Citations** | **Total Link Strength** |
| --- | --- | --- | --- | --- |
| **11285** | Lipton, Richard B. | 163 | 11,485 | 408 |
| **2464** | Buse, Dawn C. | 82 | 4,403 | 250 |
| **4909** | Dodick, David W. | 80 | 4,961 | 190 |
| **15851** | Reed, Michael L. | 43 | 2,782 | 174 |
| **18788** | Tassorelli, Cristina | 65 | 1,373 | 172 |
| **20344** | Wang, Shuu-Jiun | 63 | 1,695 | 168 |
| **7154** | Grazzi, Licia | 60 | 1,061 | 162 |
| **2996** | Cevoli, Sabina | 35 | 799 | 154 |
| **1240** | Barbanti, Piero | 33 | 843 | 141 |
| **4797** | Diener, Hans-Christoph | 45 | 2,876 | 137 |
| **17624** | Silberstein, Stephen D. | 58 | 4,405 | 121 |
| **4042** | D'Amico, Domenico | 38 | 724 | 120 |
| **128** | Adams, Aubrey Manack | 32 | 1,574 | 119 |
| **933** | Aurilia, Cinzia | 25 | 634 | 118 |
| **5630** | Fanning, Kristina M. | 28 | 1,890 | 118 |
| **871** | Ashina, Messoud | 46 | 2,566 | 112 |
| **5228** | Egeo, Gabriella | 27 | 798 | 112 |
| **6862** | Goadsby, Peter J. | 57 | 4,227 | 105 |
| **16668** | Sances, Grazia | 37 | 892 | 104 |
| **15614** | Raggi, Alberto | 29 | 404 | 94 |

**Supplementary Table 7** Institution Publication Statistics

| **ID** | **Organization** | **Documents** | | **Citations** | **Total Link Strength** |
| --- | --- | --- | --- | --- | --- |
| **127** | Albert Einstein College of Medicine | | 266 | 21074 | 684 |
| **2957** | Mayo Clinic | | 155 | 6347 | 352 |
| **4966** | University of Copenhagen | | 152 | 10928 | 276 |
| **4658** | Thomas Jefferson University | | 108 | 7691 | 223 |
| **5379** | University of Pavia | | 104 | 2677 | 203 |
| **1771** | Harvard Medical School | | 109 | 2918 | 197 |
| **5698** | Vedanta Research | | 52 | 4538 | 175 |
| **2703** | King’s College London | | 82 | 3673 | 167 |
| **3140** | Montefiore Headache Center | | 53 | 5760 | 161 |
| **4851** | Autonomous University of Barcelona | | 54 | 1574 | 160 |
| **4981** | University of Duisburg-Essen | | 68 | 2385 | 145 |
| **4578** | Taipei Veterinary General Hospital | | 77 | 1942 | 141 |
| **166** | Allergan Pharmaceuticals Inc | | 50 | 6805 | 133 |
| **1775** | Harvard University | | 107 | 7077 | 133 |
| **4939** | University of Cincinnati | | 63 | 2823 | 127 |
| **4477** | Stanford University | | 65 | 1983 | 120 |
| **167** | Allergan PLC | | 37 | 1911 | 117 |
| **202** | Amgen Inc | | 44 | 1686 | 117 |
| **3144** | Montefiore Medical Center | | 55 | 3242 | 117 |
| **5353** | University of Oslo | | 62 | 1888 | 116 |

**Supplementary Table 8** Keyword Co-Occurrence Network Analysis

| **ID** | **Keyword** | **Occurrences** | **Total Link Strength** |
| --- | --- | --- | --- |
| 5306 | prevalence | 1249 | 5582 |
| 3981 | migraine | 1463 | 4445 |
| 1983 | double-blind | 710 | 3259 |
| 2773 | headache | 861 | 3179 |
| 1891 | disability | 504 | 2495 |
| 4769 | pain | 632 | 2264 |
| 5156 | population | 429 | 2040 |
| 880 | burden | 396 | 1942 |
| 2208 | epidemiology | 402 | 1930 |
| 3124 | impact | 396 | 1909 |
| 5522 | quality-of-life | 367 | 1819 |
| 2084 | efficacy | 378 | 1777 |
| 1803 | depression | 390 | 1649 |
| 6616 | tension-type headache | 362 | 1566 |
| 1211 | chronic migraine | 348 | 1468 |
| 6960 | united-states | 271 | 1388 |
| 1917 | disorders | 306 | 1359 |
| 2222 | episodic migraine | 250 | 1353 |
| 3743 | management | 314 | 1266 |
| 1181 | chronic daily headache | 240 | 1163 |
